# Effect of Melatonin on Cognitive Function and Sleep in relation to Breast Cancer Surgery: A Randomized, Double-Blind, Placebo-Controlled Trial

**DOI:** 10.1155/2014/416531

**Published:** 2014-08-27

**Authors:** Melissa Voigt Hansen, Michael Tvilling Madsen, Lærke Toftegård Andersen, Ida Hageman, Lars Simon Rasmussen, Susanne Bokmand, Jacob Rosenberg, Ismail Gögenur

**Affiliations:** ^1^Department of Surgery, Herlev Hospital, University of Copenhagen, 2730 Herlev, Denmark; ^2^Department of Breast Surgery, Herlev Hospital, University of Copenhagen, 2730 Herlev, Denmark; ^3^Psychiatric Center Copenhagen, Rigshospitalet, University of Copenhagen, 2100 København Ø, Denmark; ^4^Department of Anaesthesia, Centre of Head and Orthopaedics, University of Copenhagen, 2100 København Ø, Denmark

## Abstract

*Background.* Sleep disturbances and cognitive dysfunction are common in patients with breast cancer. Disturbed sleep leads to poor cognitive performance and exogenous melatonin may improve sleep and attenuate cognitive dysfunction. We hypothesized that melatonin would improve sleep and cognitive function after surgery. *Methods.* This study reports secondary endpoints from a randomized, double-blind, placebo-controlled trial. Women, 30–75 years, were randomized to 6mg oral melatonin/placebo for 3 months. We assessed postoperative cognitive dysfunction (POCD) with a neuropsychological test battery, sleep with a diary, and sleep quality with VAS. *Results*. 54 patients were randomized to melatonin (*n* = 28) or placebo (*n* = 26); 11 withdrew (10 placebo, 1 melatonin, *P* = 0.002). The incidence of POCD was 0% (0/20) [95% CI 0.0%; 16.8%] in the placebo group and 0% (0/26) [95% CI 0.0%; 13.2%] in the melatonin group 2 weeks postoperatively (*P* = 1.00) and 6.3% (1/16) [95% CI 0.0%; 30.2%] in the placebo group and 0% (0/26) [95% CI 0.0%; 13.2%] in the melatonin group 12 weeks postoperatively (*P* = 0.38). Sleep efficiency was significantly greater in the melatonin group; mean difference was 4.28% [95% CI 0.57; 7.82] (*P* = 0.02). The total sleep period was significantly longer in the melatonin group; mean difference was 37.0 min [95% CI 3.6; 69.7] (*P* = 0.03). *Conclusion.* Melatonin increased sleep efficiency and total sleep time but did not affect cognitive function. The dropout rate was significantly lower in the melatonin group. This trial is registered with Clinicaltrials.gov NCT01355523.

## 1. Introduction

Sleep disturbances and cognitive dysfunction are prevalentphenomena in patients with breast cancer [1, 2]. These symptoms are often intertwined in a symptom cluster together with other common cancer-related symptoms including fatigue, depression, anxiety, and pain—all having a negative influence on overall quality of life and performance status [3–5]. Disturbed sleep and cognitive function may arise around the time of diagnosis, the time of surgery, or in the course or aftermath of adjuvant therapy [6], making the specific aetiology difficult to establish as many factors contribute to the development.

Postoperative cognitive dysfunction (POCD) is a deterioration of intellectual function postoperatively, typically presenting as impaired short-term memory or concentration [7, 8]. In the long-term, POCD has been shown to be associated with increased mortality [9] and risk of leaving the labour market prematurely [10]. Among the many pathophysiological explanations for the development of POCD, postoperative sleep disturbances are one of them [7, 11]. It is well known that overall sleep loss and sleep fragmentation may have negative cognitive consequences [12, 13] and it has previously been shown that sleep disturbances may arise in the postoperative period after surgery for breast cancer [14] and persist for months or even years [1]. Depression in itself has not been shown to be correlated with POCD in the short- or long-term postoperative period [9].

Exogenous melatonin can improve sleep quality by reducing sleep onset latency, increasing sleep efficiency, and increasing total sleep duration in healthy subjects [15] and patients with primary sleep disorders [16]. Furthermore, some studies have shown that melatonin can influence cognition positively in healthy men exposed to a stress-test and in adults with mild cognitive impairment [17–19]. Therefore, we hypothesized that melatonin would have a beneficial effect on cognitive function and sleep after breast cancer surgery with less cognitive disturbances and better sleep in the patients treated with melatonin. Our primary aim was to evaluate the effect of melatonin on cognitive function 2 weeks postoperatively.

## 2. Methods

The reporting of this study was conducted according to The CONSORT statement [20].

### 2.1. Design Overview

The MELODY trial was a randomized (1 : 1), double-blind, placebo-controlled trial [21] that primarily sought to investigate depressive symptoms as reported in another paper [22]. The study was approved by the local Ethics Committee (H-4-2011-007), the Danish Medicines Agency (EudraCT no. 2010-022460-12) and the Danish Data Protection Agency (2007-58-0015/HEH.750.89-12). The trial was registered on https://www.clinicaltrials.gov/(NCT01355523) before inclusion of the first patient and the Good Clinical Practice Unit at Copenhagen University Hospital monitored the trial. We obtained written informed consent from all patients.

### 2.2. Setting and Participants

The study was undertaken at the Department of Breast Surgery, Herlev Hospital, Copenhagen, Denmark. Eligible patients were women aged 30–75 years, scheduled for lumpectomy or mastectomy for breast cancer, with American Society of Anesthesiologists (ASA) classes I–III. We excluded pregnant patients and patients with signs of depression on the Major Depression Inventory (MDI). When screening patients for enrollment, we used the MDI as a diagnostic instrument and we excluded those who had mild, moderate, or severe depression using the ICD-10 criteria. Other exclusion criteria can be seen in the previously published protocol article [21]. The only change after trial commencement was that the upper limit of the age criteria was raised from 70 to 75 years on October 19, 2011, because of slow recruitment.

### 2.3. Randomization and Interventions

Approximately one week preoperatively, patients were assessed and screened for inclusion. This included administration of the Mini-Mental State Examination [23], a neuropsychological test battery [24], and the MDI [25].

On inclusion, patients were randomly assigned 1 : 1, in blocks of six, to melatonin or placebo. Patients in the intervention group received 6 mg oral melatonin daily 1 hour before bedtime for 1 week preoperatively and 12 weeks postoperatively. The randomization list was computer generated using dedicated software (http://www.randomization.com/). To assure blinding, this procedure was completed by the hospital pharmacy, which received the medicine directly from Pharma Nord (Vejle, Denmark). The pharmacy packed the melatonin/placebo in identical, sequentially numbered, sealed boxes. The participants, the health care providers, the research staff, and the investigators assessing the outcomes were all blinded to allocation and the allocation sequence by taking the sequentially numbered sealed boxes. The melatonin/placebo was supplied by Pharma Nord and the tablets and packages were physically identical.

After inclusion, patients kept a daily record of subjective sleep quality using a visual analogue scale (VAS) and a sleep diary was completed daily. A visit was scheduled 2 weeks postoperatively where patients were tested with the neuropsychological test battery and the MDI (data not reported here). During the last 10 weeks of the study, patients were assessed with the MDI twice and every 2 weeks they completed VAS and a sleep diary. At the final visit, 12 weeks postoperatively, patients were tested with the neuropsychological test battery and the MDI.

### 2.4. Outcomes and Follow-Up

The primary outcome was the incidence of depressive symptoms [22].

#### 2.4.1. Neuropsychological Testing

The patients underwent neuropsychological testing using the ISPOCD Test Battery [24] on 3 occasions: preoperatively and 2 and 12 weeks postoperatively. Seven variables from 4 neuropsychological tests (Visual Verbal Learning, Concept Shifting Task, Stroop Color-Word Test, and Letter-Digit Coding) were used in the analysis: cumulative numbers of words recalled in 3 trials and the number of words at delayed recall from the Visual Verbal Learning Test [26]; the time and number of errors in part C of the Concept Shifting Test [27]; the time and error scores from the third part of the Stroop Color-Word Interference Test [28]; and the number of correct answers from the Letter-Digit Coding Test [29].

#### 2.4.2. Sleep Diary and VAS

We assessed sleep over two different time periods. One was defined as the perioperative period, ranging from 3 days preoperatively till 8 days postoperatively and the other was defined as the long-term postoperative period, ranging from approximately 2 to 12 weeks postoperatively. Sleep diary data consisting of sleep latency (min), number of awakenings, total sleep period (TSP) (min), and sleep efficiency (%) were recorded for both time periods in the 2 groups. Sleep efficiency was defined as (TSP − sleep latency − minutes awake)/(TSP) and total sleep period as time in bed trying to sleep. Subjective sleep quality VAS measurements ranging from “best possible sleep” equivalent to 0 mm to “worst possible sleep” equivalent to 100 mm [30] were measured in the short- (daily) and long-term (every second week) postoperative period in the 2 groups.

### 2.5. Statistical Analysis

No interim analyses were done. For statistical analyses, IBM SPSS Statistics for Windows, Version 20.0 (IBM Corp., Armonk, NY, USA) was used. A *P* value of ≤0.05 was considered statistically significant. All analyses were completed as per protocol analysis.

The sample size estimation was based on a conservative estimate of the incidence of depression of 30% being decreased to 15% with melatonin [21]. The study was powered at 80% with a risk of type I error of 5% and a risk of type II error of 20%. According to this, we should include 120 patients in each group and we aimed to include 130.

The analysis of the neuropsychological test data was based on normative data from 133 females aged 40–60 years collected in another study [31]. We evaluated changes from the preoperative baseline to the 2 postoperative test sessions. In controls we calculated mean and standard deviations (SD) of these differences. The mean change in this group may be taken as estimated learning effects. For the individual patients, we compared baseline scores with the 2- and 12-week postoperative test results, subtracted the average learning effect from the changes, and divided the result by the SD of the control group to obtain a *Z* score for the 7 individual test outcomes. A large positive *Z* score indicated deterioration in cognitive function from baseline. We defined a composite *Z* score as the sum of the 7 *Z* scores and normalized this using the SD for that sum in the controls. POCD was defined as a combined *Z* score > 1.96 or a *Z* score > 1.96 in at least 2 of the 7 subtests as previously described [24]. This definition considered both general deterioration and substantial deterioration in only some tests. If patients refused or were not able to complete a specific test, the *Z* score for that test was considered a missing value. The outcomes were the incidence of POCD (yes/no) in the 2 groups at the test 2 weeks postoperatively and at follow-up 12 weeks postoperatively. For calculation of the 95% CI for the percentages of POCD http://www.graphpad.com/quickcalcs/confInterval1/ was used. Fisher's exact test was used to compare the incidence of POCD between the 2 groups. The 7 variables from the 4 neuropsychological tests are reported as median values with interquartile range (IQR).

Due to the nonnormal distribution of data, nonparametric statistics were used and data are presented as frequencies or median IQR, with the exception of the results from the bootstrapping used to analyze sleep diary data. Sleep diary data were calculated as a median for each patient in the 2 time periods. Finally, bootstrapping was used to calculate confidence intervals for the means and *P* values for the difference in means. This was performed using the “smean.cl.boot” function in the “Hmisc” library in R version 3.0.1 (R Foundation for Statistical Software, Vienna, Austria). Bootstrapping was performed with 10000 bootstrap samples and *P* values were calculated by an unpaired *t*-test.

## 3. Results

Patients were recruited fromJuly 2011 to December 2012 where the trial was terminated prematurely (*n* = 54) due to restructuring of surgery for breast cancer in the region resulting in an overall low inclusion rate and an inability to complete the trial. We tried to include other centres in other regions but this was not possible because of lack of funding.

**Figure 1:**
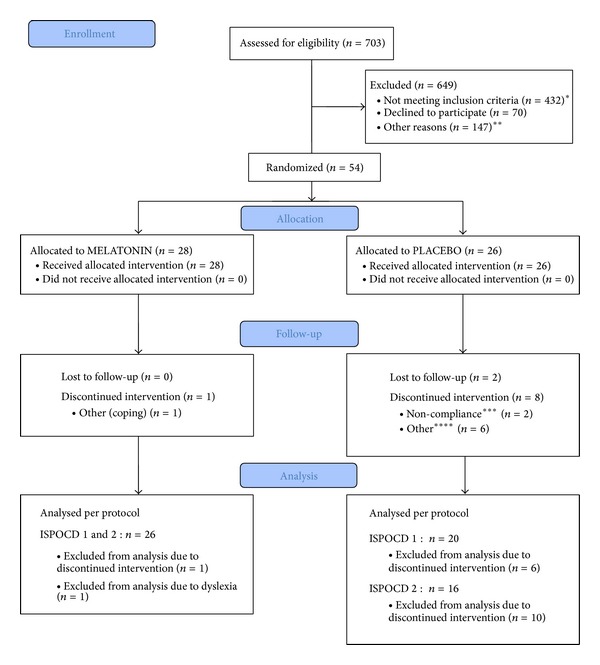
CONSORT 2010 flow diagram. (∗) age > 70/75 years = 134, DCIS or Mb. Paget = 30, preoperative chemotherapy = 51, selective serotonin reuptake inhibitors, antithrombotic drug therapy, monoaminoxidase inhibitors, calcium channel blockers = 31, epilepsy = 1, known and treated sleep apnea = 1, diabetes mellitus treated with insulin = 4, ongoing or previous medically treated depression or bipolar disorder = 44, known autoimmune disease = 9, severe kidney disease = 1, previous or other current cancer = 75, known medically treated sleep disorder = 1, daily intake of >5 units (1 unit = 8 g pure alcohol) = 3, preoperative, continuous treatment with psychopharmacological drugs of any kind, opioids, anxiolytics, or hypnotics = 35, and predicted poor compliance = 12. (∗∗) Logistics = 50, <3 days till the day of surgery = 82 and patient recruited for other trial = 15. (∗∗∗) Taken < 75% of the trial medication at visit 2. (∗∗∗∗) Adverse events = 2 (one patient had insomnia at night and sleepiness during the day and the other had headaches) and coping = 4.

The CONSORT trial profile (Figure 1) shows the screening, randomization, and follow-up of the patients; 703 patients were screened for eligibility, 649 were excluded, and the remaining 54 were randomized to either melatonin (*n* = 28) or placebo (*n* = 26). Baseline, perioperative and demographic characteristics were similar between the two groups (Table 1), although median duration of surgery was 92 and 125 min and duration of anaesthesia was 155 and 190 min in the melatonin and placebo groups, respectively.

Of the patients, 11 withdrew from the study: 10 in the placebo group—2 lost to follow-up, 2 due to noncompliance, 2 due to adverse events, and 4 due to not being able to cope with participating in the trial—and one in the melatonin group due to not being able to cope with participating in the trial (*P* = 0.002).

### 3.1. Postoperative Cognitive Dysfunction

The first neuropsychological test after baseline was completed at a median of 14 (IQR 12–14) days after surgery and the second at a median of 85 (IQR 83–90) days after surgery. POCD could be assessed in 46 patients at the first postoperative test after baseline (a combined *Z* score could not be calculated in 1 patient due to dyslexia and 7 patients due to discontinued intervention) and in 42 patients at the test at 12 weeks postoperatively (4 more discontinued intervention). The incidence of POCD was 0% (0/20) [95% confidence intervals (CI) 0.0%; 16.8%] in the placebo group and 0% (0/26) [95% CI 0.0%; 13.2%] in the melatonin group 2 weeks postoperatively (*P* = 1.00) and 6.3% (1/16) [95% CI 0.0%; 30.2%] in the placebo group and 0% (0/26) [95% CI 0.0%; 13.2%] in the melatonin group 12 weeks postoperatively (*P* = 0.38) (Table 2). Data for the 7 variables from the 4 neuropsychological tests are presented in Table 3.

### 3.2. Sleep Diary (Table 4)

In the short-term perioperative period sleep efficiency was significantly greater in the melatonin group compared with placebo with a mean difference of 4.28% [95% CI 0.57; 7.82] (*P* = 0.02). Other variables in the short-term perioperative period did not show any significant differences. In the long-term postoperative period the total sleep period was significantly greater in the melatonin group compared with placebo with a mean difference of 37.0 min [95% CI 3.6; 69.7] (*P* = 0.03).

### 3.3. Sleep Quality (Table 4)

Subjective sleep quality assessed by VAS between the melatonin and placebo group was not significantly different in the short-term perioperative (mean difference −4.71 mm [95% CI −14.63; 5.45] (*P* = 0.37)) or long-term postoperative period (mean difference −0.78 mm [95% CI −10.20; 8.48] (*P* = 0.87)).

### 3.4. Side Effects

Common side effects in the melatonin group were dizziness (14%), headache (10%), and paresthesia in the mouth region, arms, or legs (10%). Headache (27%), difficulty falling asleep (13%), and nausea (13%) were the most common side effects in the placebo group. Of the patients in the melatonin group, 56% (15/27) experienced at least one side effect, whereas this applied to 50% (12/24) in the placebo group (*P* = 0.78).

## 4. Discussion

To our knowledge this is the first study to investigate the effect of oral bedtime melatonin on cognitive function and sleep in patients undergoing surgery for breast cancer. Cognitive dysfunction was not detected as a significant problem but the sample size does not allow firm conclusions. Melatonin increased sleep efficiency in the short-term postoperative period and increased total sleep period in the long-term postoperative period compared to placebo. However, there were no differences in subjectively assessed sleep quality at any time between the 2 groups. No differences in side effects were found.

We anticipated that we would find a higher incidence of POCD, especially at the first measurement, as previous studies have shown that POCD was present approximately 1 week after surgery in 7–41% of patients depending on age, magnitude of surgery, type of anaesthesia, and type of hospitalization [9, 24, 31–33]. An overall explanation could be the use of a more modern anaesthetic regimen, multimodal pain treatment, and shorter length of hospital stay than in the years these studies enrolled patients (1994–2002). Our finding of a very low incidence of POCD may allude that this postoperative complication is not as prominent as it was in the past, but this needs to be confirmed in larger studies and in other categories of surgical patients.

Another explanation for the low incidence could be that the preoperative assessment underestimated cognitive function due to the negative influence of the psychological aspect on performance in neuropsychological tests [34], as the testing was done close to or on the actual day where the patients were diagnosed with cancer and also close to their day of surgery.

Furthermore no general consensus exists on the optimal duration of the intervals between surgery and test sessions [34]. We evaluated POCD at a median of 14 and 85 days after surgery, where other studies [9, 24, 31–33] used the same test battery earlier (5–9 days after surgery) or later (96–117 days). Possibly we tested too late to pick up the cognitive dysfunction in the immediate postoperative period and cognitive improvement had already occurred.

Another study [5] in a population of patients with breast cancer did not find objective cognitive dysfunction either, at a mean of 34.5 (range 19–75) days after surgery using the same neuropsychological test battery as in our study, although interestingly they found that the women perceived themselves as cognitively impaired, compared to healthy controls. In addition, it has been reviewed that there is a lack of a relationship between objective and subjective cognitive dysfunction in breast cancer patients [35]. This emphasizes that even though we did not detect any objective difference in cognitive decline between the two groups, it could possibly be present subjectively and thereby have clinical relevance.

Sleep disturbances were less common than anticipated in the postoperative period [36] and patients in both groups showed normal sleep on all 4 parameters when compared to a population of healthy subjects also using a sleep diary [37]. This could be yet an explanation for the low incidence of POCD, as we had hypothesized that sleep disturbances were one of the contributing factors to the development of POCD [7, 11].

The well-known effects of melatonin on sleep with an increased sleep efficiency and total sleep duration measured by polysomnography or actigraphy [15] were confirmed by our study. We showed these two changes in measurable sleep parameters, which we believe are large enough in size to be clinically relevant, although we did not find a corresponding change in subjective sleep quality (VAS). Another study [30] investigating the effect of 5 mg melatonin for 3 nights postoperatively found a decrease in sleep latency in the melatonin group and this was not associated with improved subjective sleep quality (VAS) either. A possible explanation for these findings could be that the measurement of subjective sleep quality by VAS is too imprecise to detect a difference. It could be valuable to investigate what the minimal clinically relevant difference is on VAS for sleep quality in future studies.

Based on an estimate of POCD of 30% in middle-aged patients 6 days after major surgery [9] and an expectation of a reduction to 15% with melatonin treatment, we should have included around 260 patients, which corresponds to the original sample size calculation done on the incidence of depression [21]. Our study was not completed and together with a possible underestimation of cognitive function at baseline and the later timing of the first postoperative test, these major limitations could explain the negative findings. However, with the timing of our tests we found a very low incidence of cognitive dysfunction, which means that we would most likely not have been able to find a difference, even in 260 patients. We still believe that future studies are warranted and that melatonin could be a potentially useful treatment for POCD, despite the negative results of this study, most likely due to the very low incidence of POCD.

The external validity of our study is limited in its present form as 61% (432/703) of the patients assessed for eligibility were excluded due to exclusion criteria and we could overall only include about 8% (54/703) of those assessed for eligibility. To improve the external validity, future studies should employ less strict exclusion criteria.

Regarding the internal validity of our study, it was highly influenced by the distribution of drop-outs, 10 : 1 (placebo : melatonin). Due to the high rate of drop-outs, and therefore a missing data problem, which we assessed could not be sufficiently solved by any type of imputation, we chose not to complete intention-to-treat analyses. Lost to follow-up or discontinuation of the intervention can be a possible cause of bias. This skewness in the distribution of drop-outs is interesting and can possibly be explained by a significantly lower risk of developing depressive symptoms in the melatonin group [22]. Patients in the placebo group might have dropped out due to more depressive symptoms, contributing to overall reduced mental well-being and possibly reduced cognitive function and/or poor sleep, although the latter was not quantifiable by the measurement instruments used in this study. This could contribute to an underestimation of cognitive function and sleep disturbances in the placebo group. Future studies are needed to determine whether melatonin administration is associated with improved mental and physical functioning in cancer patients, giving them the ability to complete participation in a trial.

In conclusion, melatonin increased sleep efficiency and total sleep time after breast cancer surgery, but we did not demonstrate an effect of melatonin on cognitive function after surgery. Furthermore, the drop-out rate was significantly lower in the melatonin group than in the placebo group.

## Figures and Tables

**Table 1:** Baseline, perioperative, and demographic characteristics.

Patient characteristics	MELATONIN (*n* = 28)	PLACEBO (*n* = 26)
Age (years)	51 (46–66)	60 (49–68)
BMI (kg/m^2^)	24.3 (21.2–27.1)	24.2 (21.3–26.3)
Menopausal status (pre/post)	14/14	10/16
ASA physical status class I/II/III	19/8/1	17/7/0
Level of education		
Finished grade 8	1	1
Finished grade 9/10	5	5
Graduate from high school	3	3
Some college	7	10
Bachelor's degree	8	6
Master's degree	4	1
Job status		
Student	0	0
Employed	19	14
Unemployed	1	0
Sick-leave	1	0
Disability pension	0	1
Retired	7	11
Relationship status		
Married or living as married	21	22
Divorced	3	4
Widowed	1	0
Single	3	0
Household income		
Not given	0	1
<300.000 kr.	6	5
300.000–600.000 kr.	11	10
600.000–900.000 kr.	5	8
>900.000 kr	6	3
Smoker (YES/NO/EX-SMOKER)	5/16/7	5/14/7
Type of surgery		
Mastectomy + axillary dissection +/− SN	1	3
Mastectomy + SN	4	3
Lumpectomy + axillary dissection +/− SN	6	6
Lumpectomy + SN	16	12
Lumpectomy → mastectomy + SN	1	0
Bilateral lumpectomy + SN + axillary dissection	0	2
Surgery duration (min)	92 (74.5–125)	125 (104.5–156.5)
Anaesthesia duration (min)	155 (130–187)	190 (155–225)
Oncological treatment^‡^		
None	3	3
Radiation	5	6
Chemotherapy	16	7
Chemotherapy + radiation	0	0
Radiation × 1 only	3	1
Antihormone^‡^ (no/yes (femar/letrozol/tamoxifen))	20/7	10/7
MDI baseline	6.5 (4–12.5)	7 (4.5–10)

Values are frequencies or median (25–75% IQR).

ASA: American society of anesthesiologists

BMI: body mass index

SN: sentinel node.

^‡^Only registered as which oncological treatment and antihormone therapy (if any at all) the patients received during their participation in the MELODY trial.

**Table 2:** Incidence of postoperative cognitive dysfunction (POCD) for melatonin versus placebo.

Variable	Placebo 14 (12.75–14) days after surgery	Melatonin 13 (11–15) days after surgery	*P*	Placebo 84 (80–86) days after surgery	Melatonin 85 (83–91) days after surgery	*P*
POCD (yes/no)	0/20	0/26	1.00	1/15	0/26	0.38
	0% [0.0%; 16.8%]	0% [0%; 13.2%]		6.3% [0.0%; 30.2%]	0% [0.0%; 13.2%]	

Calculations based on normative data from 133 females aged 40–60 years [30].

*P* value: Fisher's test.

Neuropsychological testing in median (IQR) days after surgery.

Values are number of patients, percentages, and 95% confidence intervals.

**Table 3:** The 7 variables of the 4 neuropsychological tests.

	Baseline MEL	Baseline PLC	Test 1 MEL	Test 1 PLC	Test 2 MEL	Test 2 PLC
Visual Verbal Learning, cumulated recall	30 (25–33)	27 (25–30)	31 (27–33.25)	30 (27–31.75)	32.5 (29–37.25)	28.5 (23.25–30)
Visual Verbal Learning, delayed recall	11 (9–13)	10 (7.5–11)	11 (10–13.25)	11 (8–12)	11 (9.75–12.25)	9 (8–10)
Concept Shifting Test, time	27.34 (22.62–34.87)	32.91 (28.11–39.51)	26.25 (22.85–34.13)	33.5 (27.17–36.29)	32.16 (23.06–37.78)	30.86 (27.51–38.29)
Concept Shifting, error score	0 (0-0)	0 (0-0)	0 (0-0)	0 (0-0)	0 (0-0)	0 (0-0)
Stroop Color-Word Interference, time	40.20 (33.16–46.93)	44.41 (39.95–52.75)	36.37 (28.53–43.47)	39.96 (34.81–43.69)	35.41 (29.88–43.00)	38.40 (33.49–45.63)
Stroop Color-Word Interference, error score	0 (0-0)	0 (0–0.5)	0 (0-0)	0 (0-0)	0 (0-0)	0 (0–0.75)
Letter-Digit Coding	35 (29.5–38.75)	33 (29–37)	38 (33–40)	36 (30.75–38.75)	36 (22–41)	39 (35–41)

Values are median (IQR).

Data are presented as number of words, time in seconds/milliseconds, or number of errors.

MEL: melatonin, PLC: placebo.

**Table 4:** Sleep latency, number of awakenings, total sleep period, sleep efficiency, and VAS sleep quality for the melatonin versus placebo in the short-term perioperative and the long-term postoperative period.

	Short-term perioperative period∗			Long-term postoperative period∗∗		
	Mean difference MEL-PLC	95% CI	*P* value	Mean difference MEL-PLC	95% CI	*P* value
Sleep latency (min)	−3.78	[−9.63; 1.71]	0.18	0.01	[−10.76; 13.63]	0.95
Number of awakenings	−0.29	[−0.85; 0.33]	0.34	−0.26	[−0.91; 0.38]	0.42
Total sleep period (min)	2.3	[−28.3; 32.5]	0.89	37.0	[3.6; 69.7]	0.03
Sleep efficiency (%)	4.28	[0.57; 7.82]	0.02	0.6	[−3.9; 4.8]	0.76
VAS sleep quality	−4.71	[−14.63; 5.45]	0.37	−0.78	[−10.20; 8.48]	0.87

Sleep diary data were calculated as a median for each patient in the 2 time periods. Bootstrapping was used to calculate confidence intervals for the means and *P* values for the difference in means. This was performed using the “smean.cl.boot” function in the “Hmisc” library in R version 3.0.1 (R Foundation for Statistical Software, Vienna, Austria).

MEL: melatonin.

PLC: placebo.

CI: confidence interval.

Sleep efficiency = (TSP − latency − minutes awake)/(TSP)%.

VAS: visual analogue scale.

∗ includes only patients who completed the whole short-term sleep diary (3 days preoperatively to 8 days postoperatively) MEL, *n* = 27, and PLC, *n* = 20.

∗∗ includes only patients who completed the whole long-term sleep diary (2 to 12 weeks postoperatively) MEL, *n* = 27, and PLC, *n* = 16.
